# Time-Differenced Carrier Phase Technique for Precise Velocity Estimation on an Android Smartphone

**DOI:** 10.3390/s22218514

**Published:** 2022-11-04

**Authors:** Antonio Angrisano, Giovanni Cappello, Silvio Del Pizzo, Salvatore Gaglione

**Affiliations:** 1 Department of Engineering, Messina University, Contrada di Dio, Sant’Agata, 98166 Messina, Italy; 2 Department of Sciences and Technologies, “Parthenope” University of Naples, Centro Direzionale Isola C4, 80143 Naples, Italy

**Keywords:** TDCP, smartphone, carrier phase, cycle slips, RAIM, GNSS

## Abstract

GNSS (Global Navigation Satellite System) receivers are not only able to accurately determine position, but also velocity, knowledge of which could be important in several applications. The most adopted technique for velocity estimation exploits the Doppler shift due to the relative motion between the signal source and the receiver. Alternatively, the TDCP (Time-Differenced Carrier Phase) technique, based on the differences between consecutive carrier-phase measurements, can be used. TDCP is theoretically able to achieve better performance compared with the Doppler-based approach, exploiting the high precision of a carrier-phase observable, and without suffering the ambiguity issue. The main objective of this study is to analyze TDCP performance on a smartphone GNSS chip. Smartphones GNSS receivers are usually characterized by noisy observables owing to the low quality of the antenna used; it is, therefore, interesting to compare the smartphone TDCP performance with that of the Doppler-based technique. To evaluate the benefits that TDCP can provide, especially in terms of the smartphone chip, these two approaches to velocity determination are compared using three different devices: a Novatel geodetic receiver, a u-blox multi-frequency receiver, and a Xiaomi Mi8 smartphone. The results demonstrate a performance degradation in the smartphone GNSS chip when TDCP is used, compared with the performance of higher-grade receivers. In fact, the Xiaomi Mi8 maximum errors are greater than those of the Novatel geodetic receiver, but they are still acceptable as they do not exceed 6 cm/s, making the TDCP technique a valid approach for advanced algorithms; indeed, TDCP velocity demonstrates a few mm/s accuracy with a smartphone. The application of a RAIM algorithm enables error reduction and the achievement of reliable information; the obtained solution reliability is about 89%.

## 1. Introduction

GNSS provide the three-dimensional position, velocity, and UTC time synchronization, worldwide and in every meteorological condition. The main component of GNSS is represented by GPS (Global Positioning System), which is fully operational and comprises MEO (Medium Earth Orbit) satellites orbiting the Earth twice a day, and a global network of ground stations tracking the satellites, analyzing the signals, and sending commands and data to the system. The main GPS (and GNSS) application is real-time navigation, which can be performed with low-cost receivers; however, it can also be used for topographical and geodetical applications, such as the monitoring of structures, plate tectonics monitoring, a survey of points on large-scale cartography, and so on [1,2,3,4,5].

The main interest of this study is real-time navigation. Indeed, nowadays, smart devices, such as smartphones, smartwatches, and tablets, are largely equipped with GNSS chips, allowing pedestrian navigation, sports tracking, and location-based services. These applications need accurate and reliable positioning that the GNSS chips are not always able to provide, mainly because of their small size, low-cost, and linear-polarized antenna [6,7,8]. In this context, advanced processing algorithms are necessary, and an accurate estimation of velocity could play a key role. The TDCP (Time-Differenced Carrier Phase) technique is able to provide mm/s order accuracy for high-grade receivers, but its performance with smart devices should be assessed in terms of accuracy and reliability. Therefore, the main objective of this work is the analysis of the TDCP-based velocity performance of a smartphone.

GNSS receivers usually estimate velocity by processing the Doppler measurement—a shift in signal frequency of the satellite–user relative motion [1].

Alternatively, velocity can be estimated by the TDCP (Time-Differenced Carrier Phase) technique [9], which employs two consecutive carrier-phase observables. The main problem with carrier-phase observables is the presence of integer ambiguity, which must be estimated. If no cycle slips occur, the ambiguity remains constant [2]; thus, differencing two consecutive measurements cancels the ambiguity [10]. This is an important aspect of TDCP, which frees it from a classical problem affecting carrier phase. The most evident benefit of TDCP is that it can provide mm/s accuracies, compared with the Doppler technique which enables estimation at the cm/s accuracy level [11]. On the other hand, TDCP is not a snapshot technique, being based on two consecutive carrier-phase measurements from the same satellite; if a loss of contact occurs on a satellite at the current or previous epoch, the TDCP observable, relative to that satellite, is unavailable. This results in a lower observable availability compared with the Doppler technique (which is a snapshot technique and does not experience this problem). An additional drawback is that the presence of cycle slip directly affects the TDCP observable so a suitable detector must be used.

TDCP was studied in several works. In [12], the authors developed an approach where TDCP aids an INS to determine the velocity of the receiver and it achieved sub-meter positional accuracies. In [13], an improved TDCP velocity-estimation approach, which was dependent on the receiver position at the current epoch and the satellite position at the current and successive epochs, was developed. A source of discontinuity in TDCP measurements is the changing of ephemerides; to overcome this limitation, in [11], a strategy based on the use of the same set of ephemerides was proposed. In [14], TDCP was used to improve pedestrian localization in an integrated scheme that included PDR (Pedestrian Dead Reckoning) and GNSS measurements.

The literature reports several studies into the development of GNSS receivers embedded into smart devices (above all smartphones); an exhaustive review of the state of art is in [15].

Several specific studies into velocity estimation and/or the TDCP technique with smart devices are found in the literature. In [16], the results from the integration of GNSS and inertial sensors of two smartphones were assessed, focusing on performance during the passage from an open sky to a more challenging environment.

In [17], a quality analysis of GNSS smartphone observations was carried out and a velocity-aided positioning approach was proposed; an analysis of velocity estimation was conducted using the carrier-phase rate and the Doppler measurements; the results showed that the carrier-phase approach was more accurate than the Doppler approach. In [18], an analysis of Doppler-based velocity estimation with smartphones was conducted for single and multi GNSS constellations, and single and dual frequencies.

In [19], GNSS pseudorange, phase, and Doppler observables were combined in order to develop a precise positioning algorithm that exploited a robust Kalman Filter to obtain the smartphone position, where a velocity term was present, the position being determined using a TDCP method.

In [20], measurements from a smartphone GNSS chip were analyzed and, owing to the presence of high noise levels, and frequent cycle slips and blunders, an advanced positioning algorithm, involving TDCP, was proposed. This research highlighted the importance of TDCP because it is crucial for advanced algorithms. A proof analysis of TDCP performance in the velocity domain using a smartphone GNSS chip is, therefore, required.

In the present work, a trial was conducted to assess the performance of the TDCP technique applied to a smartphone GNSS receiver. The considered device was a Xiaomi Mi8, equipped with a Broadcom BCM47755 chipset, able to receive L1/L5 frequencies from the GPS, GLONASS, Galileo, and BeiDou satellites. The need to investigate the TDCP performance with a smartphone arose from the typical noisiness of observations for this type of device [6,15,21,22].

A comparison was conducted with a Novatel GPS geodetic receiver, able to track L1/L2 frequencies of GPS only, and with a u-blox ZED F9P multi-band GNSS receiver, able to receive L1/L2/L5, frequencies from GPS, GLONASS, Galileo, and BeiDou [23]. Data collection was performed in an open sky context, and the velocity was estimated using both the Doppler and TDCP techniques; the data collection was static to ensure zero speed value as the reference.

The test results demonstrate that a smartphone chip provides degraded performance compared with the higher-grade devices; nevertheless, a velocity accuracy of the order of a few mm/s is attainable with TDCP. A RAIM (Receiver Autonomous Integrity Monitoring) algorithm was applied in order to reduce the effect of blunders among the measurements and to obtain reliability information about the solution.

This paper is organized as follows. In Section 2 and Section 3, the Doppler and TDCP techniques for velocity estimation are discussed, respectively. In Section 4, the RAIM concept is briefly outlined. In Section 5, the experiment and the results are described. Section 6 is dedicated to the conclusions.

## 2. Doppler-Based Velocity Estimation

In GNSS receivers, velocity estimation is usually determined by exploiting the Doppler shift in the signals transmitted by satellites. The Doppler shift is caused by the relative motion between the satellite and the user. According to the Doppler principle, the received frequency increases as the satellite approaches the receiver; it is equal to zero when the satellite reaches the minimum distance from the receiver; finally, the frequency decreases as the satellite recedes from the receiver [1]. The frequency received by the receiver, indicated as $f_{u}$, can be calculated by (1):

(1) $$f_{u}=f_{s}\left(1-\frac{{\underline{V}}_{r}\cdot \underline{e}}{c}\right)$$

where $f_{s}$ is the frequency of the signal transmitted by the satellite, $\left({\underline{V}}_{r}\cdot \underline{e}\right)$ is the dot product between the satellite–user relative velocity vector and the unit vector of the direction from satellite to user, c is the speed of light.

The satellite–receiver relative velocity is the difference between the satellite velocity, ${\underline{V}}_{s}$, and user velocity, ${\underline{V}}_{u}$:

(2) $${\underline{V}}_{r}={\underline{V}}_{s}-{\underline{V}}_{u}$$

For a generic i-th satellite, and substituting (2) in (1), it is possible to obtain:

(3) $$f_{u_{i}}=f_{s_{i}}\left\{1-\frac{1}{c}\left[\left(\underline{V_{s_{i}}}-\underline{V_{u}}\right)\cdot \underline{e}\right]\right\}$$

$f_{u_{i}}$ is the signal frequency received by the i-th satellite, and it depends on the ideal received signal frequency $f_{i}$ and the receiver clock bias drift ${\dot{\delta }t}_{u}$.

$f_{s_{i}}$ is signal frequency transmitted by the i-th satellite and generated by correcting the $f_{0}$ frequency of transmission (L1) of a term $\Delta f_{s_{i}}$ contained in the navigation message [1].

These two frequencies are expressed by (4):

(4) $$\begin{array}{c} f_{u_{i}}=f_{i}\left(1+{\dot{\delta }t}_{u}\right)\;\\ f_{s_{i}}=f_{0}+\Delta f_{s_{i}} \end{array}$$

Substituting the new expression of $f_{u_{i}}$ in (3):

$$\begin{array}{c} f_{i}\left(1+{\dot{\delta }t}_{u}\right)=f_{s_{i}}\left\{1-\frac{1}{c}\left[\left({\underline{V_{s}}}_{i}-\underline{V_{u}}\right)\cdot \underline{e}\right]\right\}\;\\ f_{i}+f_{i}{\dot{\delta }t}_{u}=f_{s_{i}}\left\{1-\frac{{\underline{V_{s}}}_{i}\cdot \underline{e}}{c}+\frac{\underline{V_{u}}\cdot \underline{e}}{c}\right\}\\ \frac{c\left(f_{i}-f_{s_{i}}\right)}{f_{s_{i}}}+\frac{{\mathrm{cf}}_{i}{\dot{\delta }t}_{u}}{f_{s}{}_{i}}=-{\underline{V_{s}}}_{i}\cdot \underline{e}+\underline{V_{u}}\cdot \underline{e}\\ \frac{c\left(f_{i}-f_{s_{i}}\right)}{f_{s_{i}}}+{\underline{V_{s}}}_{i}\cdot \underline{e}=\underline{V_{u}}\cdot \underline{e}-\frac{{\mathrm{cf}}_{i}{\dot{\delta }t}_{u}}{f_{s_{i}}} \end{array}$$

Expanding the dot product, Equation (5) is obtained:

(5) $$\frac{c\left(f_{i}-f_{s_{i}}\right)}{f_{s_{i}}}+V_{x_{s_{i}}}e_{i}^{x}+V_{y_{s_{i}}}e_{i}^{y}+V_{y_{s_{i}}}e_{i}^{z}=V_{u_{x}}e_{i}^{x}+V_{u_{y}}e_{i}^{y}+V_{u_{z}}e_{i}^{z}-\frac{{\mathrm{cf}}_{i}{\dot{\delta }t}_{u}}{f_{s_{i}}}$$

The first member of (5) contains only calculated elements, while the second member contains the unknowns of the problem, namely $\left(V_{u_{x}},V_{u_{y}},V_{u_{z}},-{\dot{\delta }t}_{u}\right)$.

Setting:

$$d_{i}=\frac{c\left(f_{i}-f_{s_{i}}\right)}{f_{s_{i}}}+V_{x_{s_{i}}}e_{i}^{x}+V_{y_{s_{i}}}e_{i}^{y}+V_{y_{s_{i}}}e_{i}^{z}$$

and considering that the ratio between $f_{i}$ and $f_{s_{i}}$ is near to 1, it is possible to neglect it in the last term $\frac{{\mathrm{cf}}_{i}{\dot{\delta }t}_{u}}{f_{s_{i}}}$ of (5), obtaining (6):

(6) $$d_{i}=V_{u_{x}}e_{i}^{x}+V_{u_{y}}e_{i}^{y}+V_{u_{z}}e_{i}^{z}-{\dot{\delta }t}_{u}$$

For a satellite number m≥4:

(7) $$\underline{d}=H\underline{v}+\underline{\varepsilon }$$

where $\underline{\varepsilon }$ is the vector containing the error sources contributions, while the remaining elements are:

$$\begin{array}{c} \underline{d}=\left[\begin{array}{c} d_{1}\\ d_{2}\\ \vdots \\ d_{m} \end{array}\right]\\ H=\left[\begin{array}{cccc} e_{1}^{x}\left(t_{2}\right) & e_{1}^{y}\left(t_{2}\right) & e_{1}^{z}\left(t_{2}\right) & 1\\ e_{2}^{x}\left(t_{2}\right) & e_{2}^{y}\left(t_{2}\right) & e_{2}^{y}\left(t_{2}\right) & 1\\ \vdots & \vdots & \vdots & \vdots \\ e_{m}^{x}\left(t_{2}\right) & e_{m}^{y}\left(t_{2}\right) & e_{m}^{z}\left(t_{2}\right) & 1 \end{array}\right]\\ \underline{V}=\left[\begin{array}{c} V_{u_{x}}\\ V_{u_{y}}\\ V_{u_{z}}\\ -c{\dot{\delta }t}_{u} \end{array}\right] \end{array}$$

The measurement Equation (7) can be solved using the Weighted Least Squares (WLS) method:

(8) $$\underline{V}={\left(H^{T}\mathrm{WH}\right)}^{-1}H^{T}W\underline{d}$$

where W is the weighting matrix,

The measurements are assumed to be uncorrelated; hence, W is a diagonal matrix. The variance of the measurements, $\sigma_{i}^{2}$, are placed on the diagonal, as shown below:

$$W=\left[\begin{array}{cccc} 1/\sigma_{1}^{2} & 0 & \cdots & 0\\ 0 & 1/\sigma_{2}^{2} & \ddots & \vdots \\ \vdots & \ddots & \ddots & 0\\ 0 & \cdots & 0 & 1/\sigma_{m}^{2} \end{array}\right]$$

All the above equations are adapted from [1].

For the present study, the diagonal elements of W are determined with a weighting model based on the carrier to noise ratio, C/N_0_, and satellite elevation (El), shown in (9) and assessed in [24]. This model was adopted after several attempts with various weighting strategies, as it provided the best results.

(9) $$\sigma_{i}^{2}=\sigma_{0}^{2}\frac{10^{\left(-0.1\;C/N_{0}{}_{i}\right)}}{\sin^{2}\left({\mathrm{El}}_{i}\right)}$$

$\sigma_{0}^{2}$ is a constant variance value.

## 3. Time-Differenced Carrier-Phase Technique

The TDCP algorithm estimates user velocity in a very accurate manner by processing the differences in two consecutive carrier-phase measurements, as long as the i-th satellite signal is received in both the current and the previous epochs [10].

**Figure 1:**
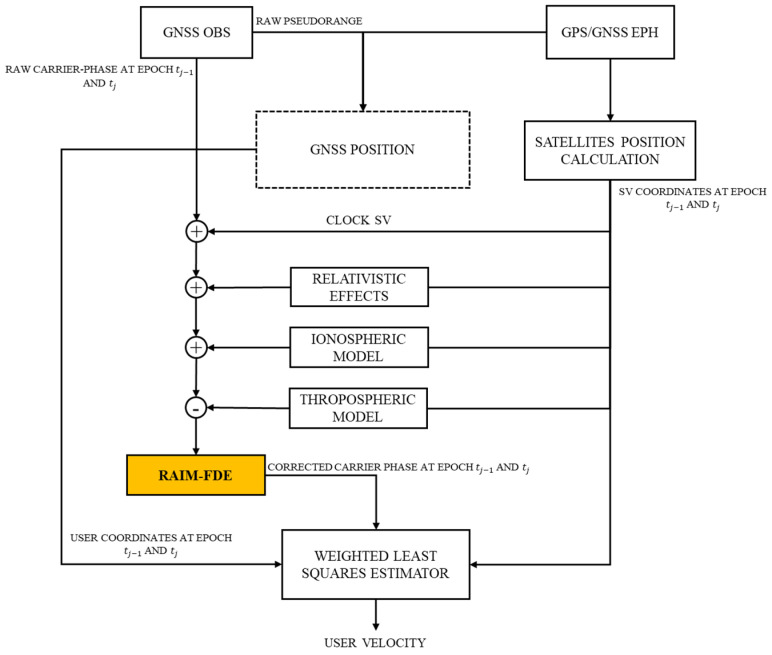
Time-Differenced Carrier Phase algorithm functional scheme (modified from [11]).

As can be seen from Figure 1, which represents how the TDCP algorithm works, user velocity is computed starting with the GNSS measurements: raw pseudorange is used to determine the GNSS position at the current epoch, referred as $t_{j}$; and two consecutive raw carrier-phase measurements at $t_{j-1}$ and $t_{j}$ epochs.

Through GPS and GNSS ephemerides, it is possible to determine the satellite’s positions and the parameters of the Klobuchar ionospheric model (this latter information is contained in the GPS navigation message [1] and explains the “GPS/GNSS EPH”-named box). It is important to note that, because the TDCP algorithm uses two consecutive carrier-phase measurements, two consecutive positions for the same satellite are needed, as also clarified from Figure 1.

The raw carrier-phase measurements are corrected from the satellite clock bias, and relativistic and atmospheric effects; just before the estimation method, the RAIM (Receiver Autonomous Integrity Monitoring) algorithm is applied to detect and exclude any carrier-phase measurements affected by blunders, especially those caused by cycle slip. RAIM is a method used to determine the integrity of the navigation solution and to protect it from an excessive horizontal position error caused by gross errors, excluding these from the navigation solution computation (Fault Detection and Exclusion (FDE) technique) [1]. RAIM theory is detailed in [25]. An application of RAIM in cycle-slip detection was assessed in [26]. In this study, RAIM is applied to the measurement set in order to exclude those blunders that can affect the accuracy of estimated velocity.

Therefore, once RAIM is applied, corrected and consistent measurements are produced. These, together with the user position at $t_{j-1}$ and $t_{j}$ consecutive epochs in the estimation method (that in this study is the WLS method), allow the user velocity to be calculated.

The carrier-phase measurements are expressed by (10):

(10) $$\lambda \phi =d+{c\delta t}_{s}-{c\delta t}_{u}+\lambda N+{\delta d}_{\mathrm{eph}}-{\delta d}_{\mathrm{iono}}+{\delta d}_{\mathrm{trop}}+\eta$$

where λ is the wavelength of the signal transmitted by the satellite, ϕ is the measured carrier phase expressed in cycles, d is the satellite–receiver geometric distance (also defined as “range”), c is the speed of light, ${\delta t}_{s}$ is the satellite clock bias, ${\delta t}_{u}$ is the receiver clock bias, N is the integer ambiguity, ${\delta d}_{\mathrm{eph}}$ is the ephemeris errors, ${\delta d}_{\mathrm{iono}}$ and ${\delta d}_{\mathrm{trop}}$ are, respectively, the ionospheric and tropospheric errors, and η is another error term which includes multipath and receiver noise.

Calculating the difference between two consecutive carrier-phase measurements, referred at epochs $t_{j}$ and $t_{j-1}$, it is possible to write (11):

(11) $$\begin{array}{cc} \lambda \Delta \phi =\lambda [\phi \left(t_{j}\right)- & \phi \left(t_{j-1}\right)]\\ & =\Delta d+{c\Delta \delta t}_{s}-{c\Delta \delta t}_{u}+{\Delta \delta d}_{\mathrm{eph}}-{\Delta \delta d}_{\mathrm{iono}}\\ & +{\Delta \delta d}_{\mathrm{trop}}+\Delta \eta \end{array}$$

Analyzing the terms of (11), it is possible to neglect ${c\Delta \delta t}_{s}$ because the corrections for satellite clock bias are contained in ephemeris data [1], so ${c\Delta \delta t}_{s}$ consists of a difference in residual errors and has a very small value. Regarding the atmospheric terms (${\Delta \delta d}_{\mathrm{iono}}$ and ${\Delta \delta d}_{\mathrm{trop}}$), these can also be neglected because they are compensated, in a single epoch, using specific algorithms: the Klobuchar model is employed to mitigate the ionospheric delay and the Saastamoinen model is used to mitigate the tropospheric delay. This means that, following the previous logic, ${\Delta \delta d}_{\mathrm{iono}}$ and ${\Delta \delta d}_{\mathrm{trop}}$ are differences in residual errors, so they can be removed. Finally, ${\Delta \delta d}_{\mathrm{eph}}$ is negligible because, assuming the quasi-constancy in time of ephemeris errors, this difference tends to a zero value.

In this way, the compensated TDCP measure equation is:

(12) $$\lambda \Delta \tilde{\phi }=\Delta d+{c\Delta \delta t}_{u}+\Delta \varepsilon$$

In (12), the term Δε contains the errors relating to multipath, receiver noise, and residuals from the other error sources.

**Figure 2:**
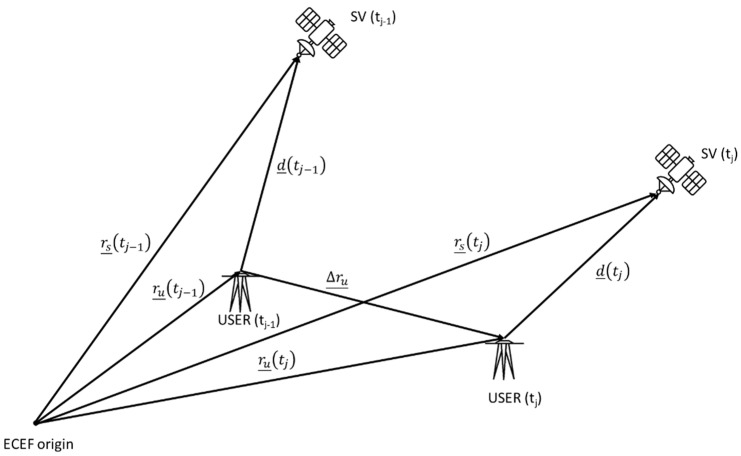
Satellite–Receiver relative geometry (modified from [9]).

From Figure 2, the satellite–receiver relative geometry can be seen, where $\underline{r_{s}}$ is the satellite position vector and $\underline{r_{u}}$ is the user position vector, both referred to in the ECEF frame, $\underline{d}$ is the geometric distance of satellite–receiver, and $\underline{\Delta r_{u}}$ is the receiver position shift.

The satellite–receiver range, varying between $t_{j-1}$ and $t_{j}$, can be formulated as follows:

(13) $$\begin{array}{c} \underline{d}\left(t_{j}\right)=\underline{e}\left(t_{j}\right)\cdot \left[\underline{r_{s}}\left(t_{j}\right)-\underline{r_{u}}\left(t_{j}\right)\right]\\ \underline{d}\left(t_{j-1}\right)=\underline{e}\left(t_{j-1}\right)\cdot \left[\underline{r_{s}}\left(t_{j-1}\right)-\underline{r_{u}}\left(t_{j-1}\right)\right] \end{array}$$

where $\underline{e}$ is the Line-Of-Sight (LOS) versor referring to $t_{j-1}$ and $t_{j}$ epochs, and “·” indicates the dot product operation.

Δd is the difference between $\underline{d}\left(t_{j}\right)$ and $\underline{d}\left(t_{j-1}\right)$:

(14) $$\begin{array}{cc} \Delta d=\underline{e}\left(t_{j}\right)\cdot [\underline{r_{s}}\left(t_{j}\right) & -\underline{r_{u}}\left(t_{j}\right)]-\underline{e}\left(t_{j-1}\right)\cdot \left[\underline{r_{s}}\left(t_{j-1}\right)-\underline{r_{u}}\left(t_{j-1}\right)\right]\\ & =\left[\underline{e}\left(t_{j}\right)\cdot \underline{r_{s}}\left(t_{j}\right)-\underline{e}\left(t_{j-1}\right)\cdot \underline{r_{s}}\left(t_{j-1}\right)\right]\\ & -\left[\underline{e}\left(t_{j}\right)\cdot \underline{r_{u}}\left(t_{j}\right)-\underline{e}\left(t_{j-1}\right)\cdot \underline{r_{u}}\left(t_{j-1}\right)\right] \end{array}$$

The receiver position at the current epoch is:

(15) $$\underline{r_{u}}\left(t_{j}\right)=\underline{r_{u}}\left(t_{j-1}\right)+\underline{\Delta r_{u}}$$

Substituting (15) in (14), obtains:

$$\begin{array}{cc} \Delta d=[\underline{e}\left(t_{j}\right)\cdot \underline{r_{s}}\left(t_{j}\right) & -\underline{e}\left(t_{j-1}\right)\cdot \underline{r_{s}}\left(t_{j-1}\right)]\\ & -\left[\underline{e}\left(t_{j}\right)\cdot \left(\underline{r_{u}}\left(t_{j-1}\right)+\underline{\Delta r_{u}}\right)-\underline{e}\left(t_{j-1}\right)\cdot \underline{r_{u}}\left(t_{j-1}\right)\right]\\ & =\left[\underline{e}\left(t_{j}\right)\cdot \underline{r_{s}}\left(t_{j}\right)-\underline{e}\left(t_{j-1}\right)\cdot \underline{r_{s}}\left(t_{j-1}\right)\right]\\ & -\left[\underline{e}\left(t_{j}\right)\cdot \underline{r_{u}}\left(t_{j-1}\right)+\underline{e}\left(t_{j}\right)\cdot \underline{\Delta r_{u}}-\underline{e}\left(t_{j-1}\right)\cdot \underline{r_{u}}\left(t_{j-1}\right)\right]\\ & =\left[\underline{e}\left(t_{j}\right)\cdot \underline{r_{s}}\left(t_{j}\right)-\underline{e}\left(t_{j-1}\right)\cdot \underline{r_{s}}\left(t_{j-1}\right)\right]\\ & -\left[\underline{e}\left(t_{j}\right)\cdot \underline{r_{u}}\left(t_{j-1}\right)-\underline{e}\left(t_{j-1}\right)\cdot \underline{r_{u}}\left(t_{j-1}\right)\right]-\left[\underline{e}\left(t_{j}\right)\cdot \underline{\Delta r_{u}}\right] \end{array}$$

Setting:

(16) $$\begin{array}{c} \Delta D=\left[\underline{e}\left(t_{j}\right)\cdot \underline{r_{s}}\left(t_{j}\right)-\underline{e}\left(t_{j-1}\right)\cdot \underline{r_{s}}\left(t_{j-1}\right)\right]\\ \Delta g=\left[\underline{e}\left(t_{j}\right)\cdot \underline{r_{u}}\left(t_{j-1}\right)-\underline{e}\left(t_{j-1}\right)\cdot \underline{r_{u}}\left(t_{j-1}\right)\right] \end{array}$$

It is possible to express Δd as follows:

(17) $$\Delta d=\Delta D-\Delta g-\left[\underline{e}\left(t_{j}\right)\cdot \underline{\Delta r_{u}}\right]$$

In (17), ΔD represents change in range, proportional to the average Doppler frequency shift due to the satellite motion, while Δg represents the relative satellite–receiver geometry change due to the orientation shift of the LOS vector.

Substituting (17) in (12):

(18) $$\lambda \Delta \tilde{\phi }=\Delta D-\Delta g-\left[\underline{e}\left(t_{j}\right)\cdot \underline{\Delta r_{u}}\right]+{c\Delta \delta t}_{u}+\Delta \varepsilon$$

Carrying ΔD and Δg terms at the first member of (9), the adjusted TDCP measurement is obtained:

$$\lambda \Delta {\tilde{\phi }}^{\mathrm{adj}}=\lambda \Delta \tilde{\phi }-\Delta D+\Delta g$$

The final expression of the TDCP measurement equation is:

(19) $$\lambda \Delta {\tilde{\phi }}^{\mathrm{adj}}=-\left[\underline{e}\left(t_{j}\right)\cdot \underline{\Delta r_{u}}\right]+{c\Delta \delta t}_{u}+\Delta \varepsilon$$

$\underline{\Delta r_{u}}$ and ${c\Delta \delta t}_{u}$ are unknown quantities which should be calculated.

For a number of available measurements *m*, at least equal to the number of unknowns, it is possible to estimate the unknowns by solving a set of equations such as (19), whose expression is:

(20) $$\underline{y}=H\underline{X}+\underline{\varepsilon }$$

where $\underline{y}$ is the vector of the measurements, $\underline{X}$ is the vector containing the unknown quantities, H is the design matrix, $\underline{\varepsilon }$ is a vector containing the measurement errors.

$$\begin{array}{c} \underline{y}=\left[\begin{array}{c} \lambda \Delta {\tilde{\phi }}_{1}{}^{\mathrm{adj}}\\ \lambda \Delta {\tilde{\phi }}_{2}{}^{\mathrm{adj}}\\ \vdots \\ \lambda \Delta {\tilde{\phi }}_{m}{}^{\mathrm{adj}} \end{array}\right]\\ \underline{x}=\left[\begin{array}{c} \Delta r_{u}^{x}\\ \Delta r_{u}^{y}\\ \Delta r_{u}^{z}\\ c\Delta \delta t_{u} \end{array}\right]\\ H=\left[\begin{array}{cccc} e_{1}^{x}\left(t_{2}\right) & e_{1}^{y}\left(t_{2}\right) & e_{1}^{z}\left(t_{2}\right) & 1\\ e_{2}^{x}\left(t_{2}\right) & e_{2}^{y}\left(t_{2}\right) & e_{2}^{y}\left(t_{2}\right) & 1\\ \vdots & \vdots & \vdots & \vdots \\ e_{m}^{x}\left(t_{2}\right) & e_{m}^{y}\left(t_{2}\right) & e_{m}^{z}\left(t_{2}\right) & 1 \end{array}\right] \end{array}$$

The WLS method can be adopted to solve Equation (20), as shown below:

(21) $$\underline{X}={\left(H^{T}\mathrm{WH}\right)}^{-1}H^{T}W\underline{y}$$

where W is the weighting matrix.

$$W=\left[\begin{array}{cccc} 1/\sigma_{1}^{2} & 0 & \cdots & 0\\ 0 & 1/\sigma_{2}^{2} & \ddots & \vdots \\ \vdots & \ddots & \ddots & 0\\ 0 & \cdots & 0 & 1/\sigma_{m}^{2} \end{array}\right]$$

The velocity can be determined as follows:

(22) $$\underline{V}=\frac{\underline{\Delta r_{u}}}{t_{j}-t_{j-1}}$$

All equations are taken from [9,11], and the W matrix elements are calculated using the same weighting strategy discussed in the previous section, scaling the constant variance value to one order of magnitude.

## 4. RAIM

Integrity refers to the capability of a navigation system to provide timely warnings to users when the system should not be used. RAIM is a user-level algorithm for solution integrity, based on a consistency check of redundant measurements [1].

**Figure 3:**
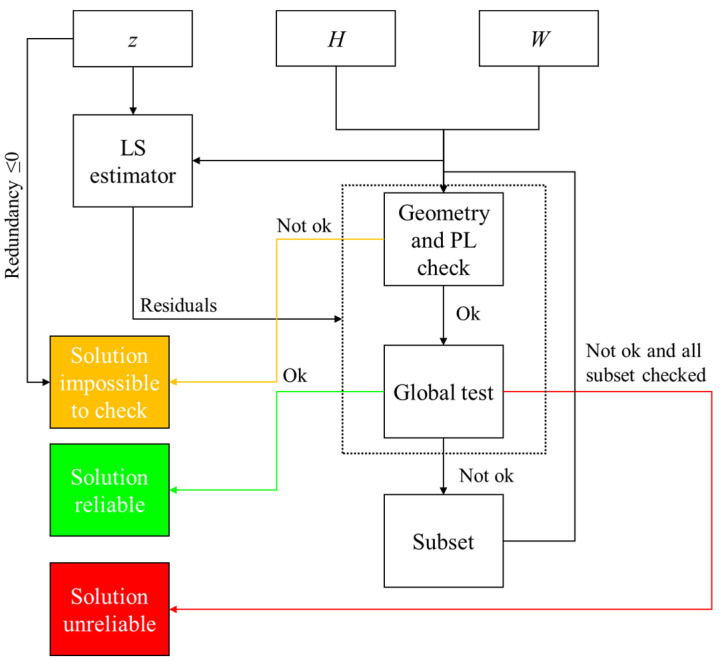
RAIM Subset algorithm.

There are several RAIM algorithms; the algorithm used in this study is the RAIM Subset, whose functional scheme is shown in Figure 3. The inputs of this algorithm are the residual vector, the design matrix *H*, and the weighting matrix *W*.

Before RAIM application, a check on satellite geometry and protection levels (PL) is carried out and, if this test fails, the solution is declared impossible to check, as indicated in the yellow box. The satellite geometry check consists in comparing DOP parameters against suitable thresholds: if DOP values exceed the threshold, the observation geometry is considered “bad” [1], and the solution is declared impossible to check. Similarly, if Horizontal and Vertical Protection Levels (HPL and VPL, respectively), largely detailed in [27], exceed the limit values named the Horizontal Alert Limit and Vertical Alert Limit (HAL and VAL, respectively), the measurement set is considered impossible to check.

After the Geometry and PL checks, a Global Test (GT) is carried out to verify the self-consistency of the measurement set. In this phase, a decision variable *D* is computed by (23):

(23) $$\underline{D}={\underline{r}}^{T}W\underline{r}$$

where *r * is the residual vector, obtained by:

(24) $$\underline{r}=\underline{z}-H\underline{\hat{x}}$$

where $\underline{z}$ is the measurement vector and $\underline{\hat{x}}$ is the state vector, estimated using the WLS method.

$\underline{D}$ is assumed to follow a $\chi^{2}$ distribution with $\left(m-n\right)$ degrees of freedom (or redundancy), defined as the difference between the number of measurements *m* and the state dimension *n*, and it is compared with the threshold $T_{g}$:

(25) $$T_{g}=\chi_{1-P_{FA},\left(m-n\right)}^{2}$$

where $\left(1-P_{FA}\right)$ indicates the abscissa value of a $\chi^{2}$ distribution of $\left(m-n\right)$ degrees of freedom. $P_{FA}$ is the probability of a false alarm and, in this experiment, is fixed at 0.1%.

In subset testing, the global test is applied to all possible measurement subsets until a subset is found from which the supposed blunders are excluded. This is done by searching for a subset that most clearly passes the global test, i.e., which satisfies its self-consistency test with the smallest test statistic. The subset with the smallest test statistic below the threshold, and the largest number of measurements, is chosen to provide the best position solution [28].

## 5. Test and Results

The main objective of this study was to analyze the velocity estimation performance of a smartphone GNSS; in particular, the TDCP technique was considered. The smartphone Xiaomi Mi8 was used for the test; the device is equipped with a Broadcom BCM47755 chipset, and is able to receive L1/E1 and L5/E5 signal frequencies from the GPS, GLONASS, Galileo, and BeiDou constellations [29].

For comparison, a geodetic receiver and a high-sensitivity receiver were also considered, specifically a Novatel Geodetic GPS receiver and a u-blox ZED-F9P GNSS receiver. The former device is a double-frequency GPS receiver; the latter is a multi-frequency GNSS receiver. In this work, only GPS measurements were analyzed.

**Figure 4:**
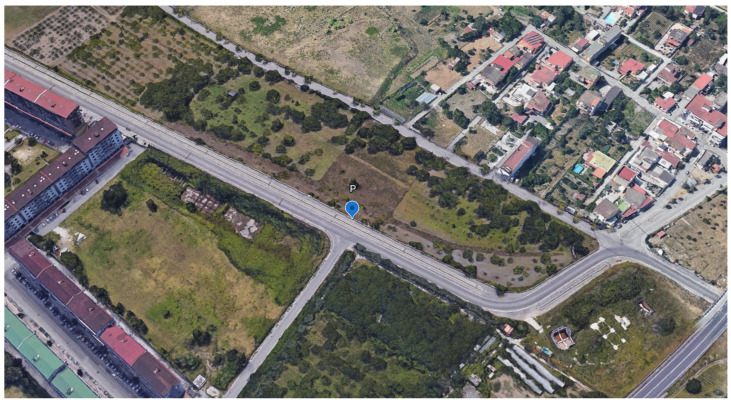
Test location.

For this experiment, all the devices simultaneously acquired about 22 min of raw GPS data at L1 frequency in static mode in order to compare the estimated velocity with a reference of zero speed value. The data were collected on 24 April 2022 in a rural area in the suburbs of Naples (Figure 4).

**Figure 5:**
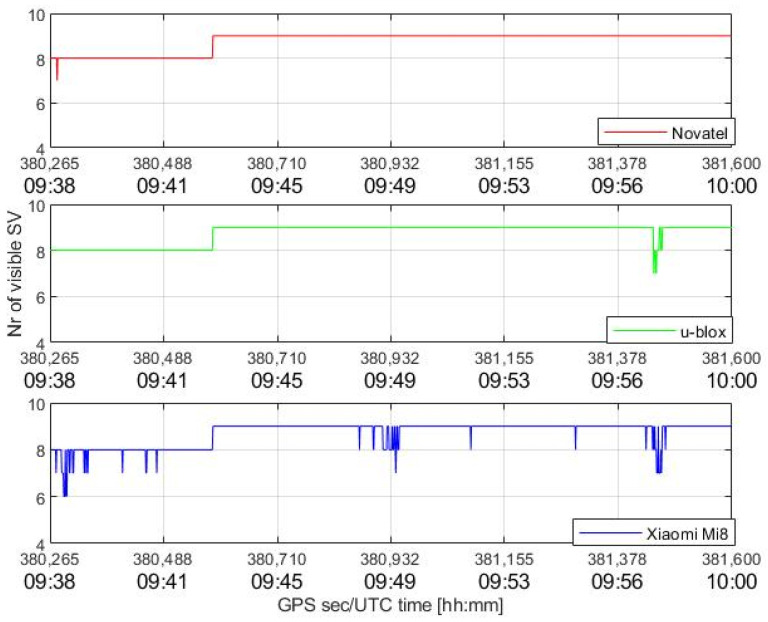
Satellite visibility for each device.

The test scenario was open sky, as can be seen in Figure 4; satellite visibility was, therefore, good during the entire session, as shown in Figure 5.

Figure 5 shows an overview of the number of visible GPS satellites for each device; visible satellites were well above the minimum number of four satellites necessary for the estimation of the unknowns. Initially, there were eight visible satellites for each device, and then nine from about 09:42. The smartphone visibility was the least stable, as indicated by the frequent drops; however, the number of visible satellites from the Mi8 was never less than 6.

**Figure 6:**
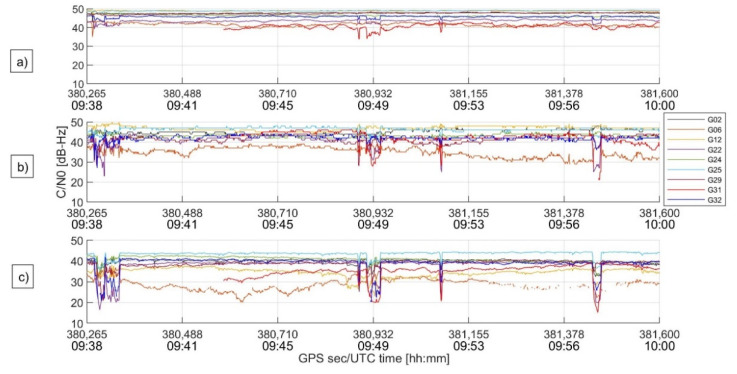
C/N_0_ behavior for each visible satellite.

Figure 6 shows the C/N_0_ behavior for each device. It can be seen that this ratio decreases in the devices in the following order: Novatel geodetic receiver (a), u-blox receiver (b), and Mi8 smartphone (c). For the geodetic receiver, the C/N_0_ values are mainly between 40 and 50 dB-Hz; for the high-sensitivity device, the values are mainly between 30 and 50 dB-Hz; finally, for the smartphone, the values are between 20 and 45 dB-Hz.

This is a clear indication that the observations collected by the smartphone are noisier than the other devices.

At three moments, around 9:39, 9:49, and 9:58, a drop in C/N_0_ values, probably related to unknown interferences, is evident; the main effects are on the u-blox and, most of all, on the Xiaomi, and they correspond to the decrease in the available measurements in Figure 5.

In order to assess the performance of the smartphone GNSS chip for velocity estimation, Doppler-derived and TDCP-derived velocities were considered.

Collected observables were processed with PANG-NAV, a MATLAB^®^ tool developed by PANG (PArthenope Navigation Group) able to obtain a PVT (Position, Velocity, Timing) solution and, in the presence of a reference, to perform the error analysis. Further details about PANG-NAV are provided in [30]. PANG-NAV is able to apply the FDE-RAIM (Fault Detection and Exclusion-RAIM) algorithm.

Table 1 and Table 2 summarize the experimental results, which were obtained without and with RAIM, respectively. The analyzed configurations are reported in the first and second columns, indicating the device used (Novatel, u-blox, or Xiaomi) and technique (Doppler-based or TDCP-based velocity estimation), respectively. The considered figures of merits are the mean, the root mean square (RMS), and the maximum errors for horizontal (H) and vertical (U) directions. The last columns of Table 1 and Table 2 report the solution availability and the solution reliability, respectively. Solution availability indicates percentage of available solutions, i.e., when the number of measurements is sufficient for velocity computation. Solution reliability is the percentage of reliable solutions (on the total of potential epochs) according to RAIM.

Table 1 shows that the theoretical performance of Doppler-based and TDCP-based velocity estimation are confirmed for the geodetic receiver, with an accuracy of cm/s and mm/s order, respectively. A degradation is evident for the high-sensitivity and smartphone receivers, with larger values for each considered figure of merit. In particular, the presence of anomalous measurements is remarkable in the TDCP results for the u-blox, characterized by a maximum error of 4 dm/s (horizontal) and 1.3 m/s (vertical). The application of RAIM is, therefore, necessary for TDCP in order to identify and exclude anomalous measurements, often related to cycle slips, and to obtain a reliable solution. Finally, the solution availability is 100% for the Doppler approach for all the considered devices; the 99.9% value for TDCP case is simply due to the impossibility of differencing consecutive carrier phases at the first epoch.

From Table 2, it can be observed that TDCP provides better velocity accuracy compared with the Doppler technique for all the devices. On the other hand, the reliability percentage obtained with TDCP is lower compared with the Doppler-based application; this happens mainly because TDCP is prone to the issue of cycle slips, which are considered by RAIM as blunders.

As expected, the Novatel Geodetic Receiver, which was the highest-grade device used, provides the best performance for all the considered figures of merits. TDCP RMS errors are one order of magnitude smaller than those of the Doppler approach; the reliability percentage of the Doppler solution is 100%, while it is 95.6% for TDCP, because a number of anomalous measurements were detected by RAIM, which flagged about 50 epochs as “unreliable”.

The u-blox receiver with the TDCP technique also provided accuracy at the mm/s level. This configuration evidently benefits from RAIM application. Indeed, horizontal and vertical maximum errors decrease from 4.2 dm/s to 2.4 cm/s and from 1.3 m/s to 7.6 cm/s, respectively; the benefits are also clear in terms of RMS and mean errors. The reliable availability of this configuration is 96.4%.

For the Mi8 GNSS chip, the TDCP technique provided better performance compared with the Doppler-based technique, as it did for the other considered devices. The TDCP performance was good, with RMS horizontal and vertical errors equal to 7 and 16 mm/s, respectively, and maximum horizontal and vertical errors less than 6 cm/s and 2 dm/s, respectively. The solution reliability is lower than the other two devices; indeed, it is 89.5%. This confirms that, as previously discussed in Section 1, the smartphone observables are noisier, and the cycle slip more frequent compared with the other devices.

**Figure 7:**
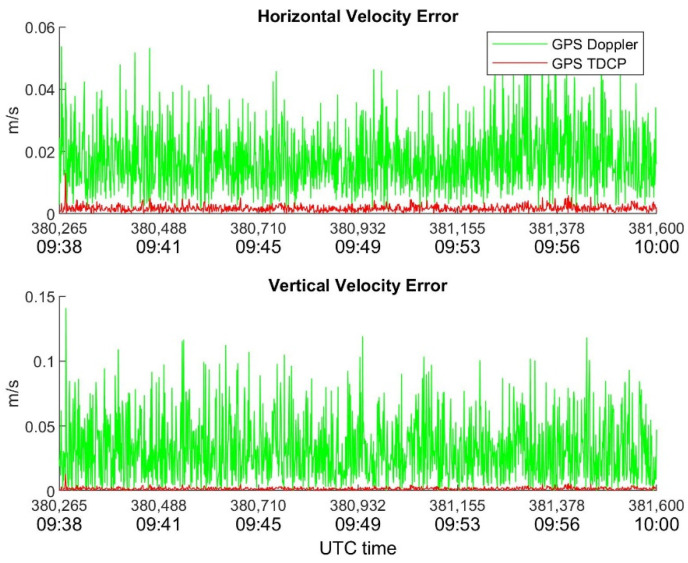
Novatel horizontal and vertical velocity errors as function of time. RAIM is applied.

**Figure 8:**
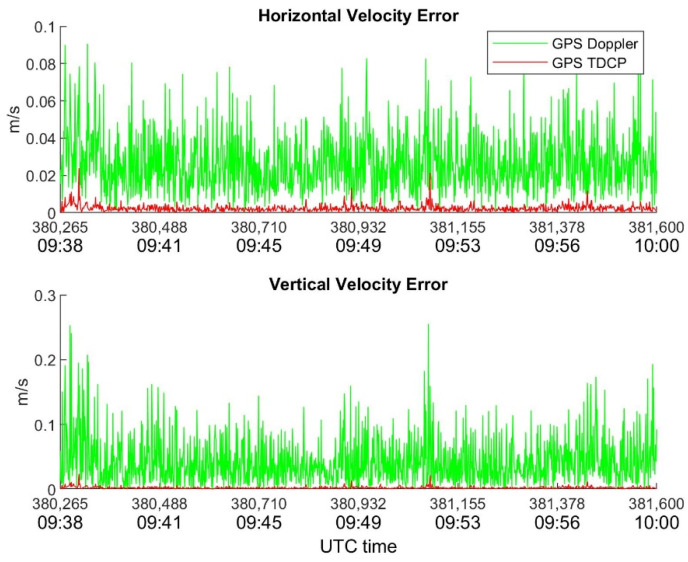
u-blox horizontal and vertical velocity errors as function of time. RAIM is applied.

**Figure 9:**
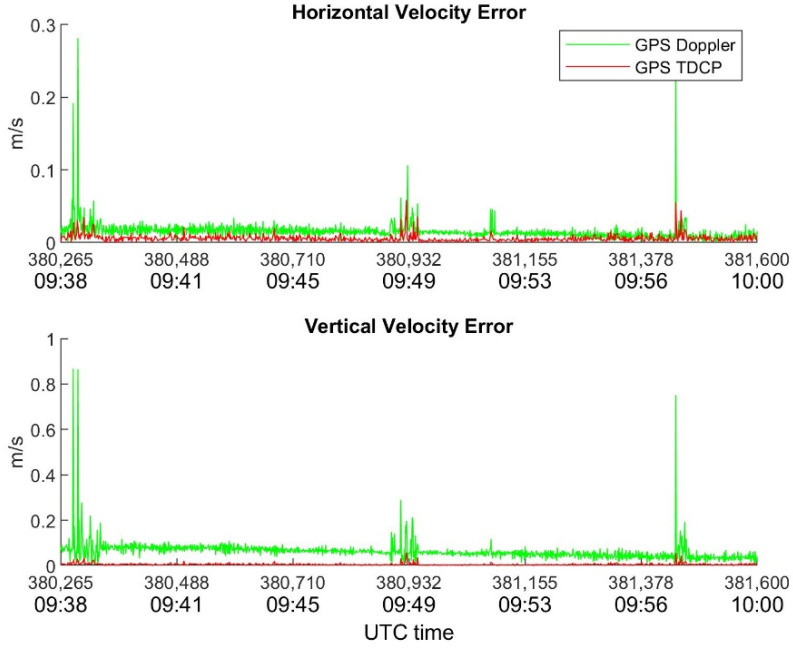
Xiaomi Mi8 horizontal and vertical velocity errors as function of time. RAIM is applied.

In order to highlight the benefits of TDCP compared with Doppler for velocity estimation, Figure 7, Figure 8 and Figure 9 show the behavior of horizontal and vertical velocity errors for each device. These figures were obtained by applying RAIM.

It is interesting to note that several spikes are present in Figure 9 which refers to the Xiaomi device; such sudden increases in error occur in both the Doppler and TDCP cases and are related to the sudden drops in visibility and C/N_0_ shown in Figure 5 and Figure 6, respectively.

**Figure 10:**
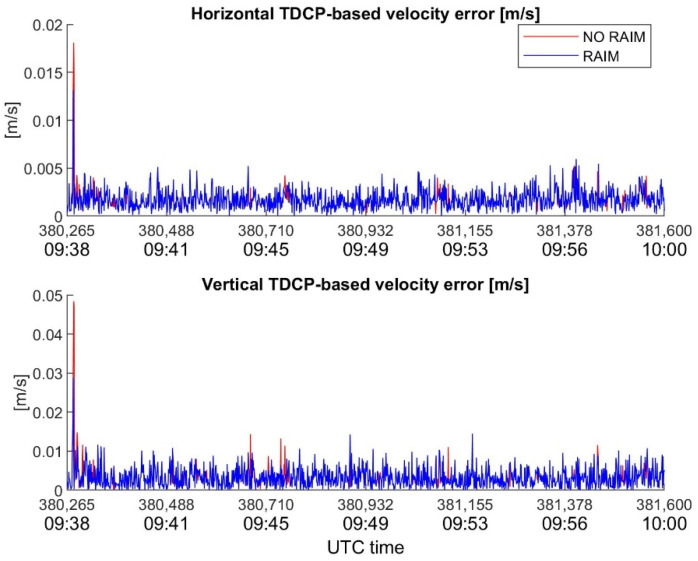
Novatel horizontal and vertical TDCP-based velocity errors as function of time, with and without RAIM application.

**Figure 11:**
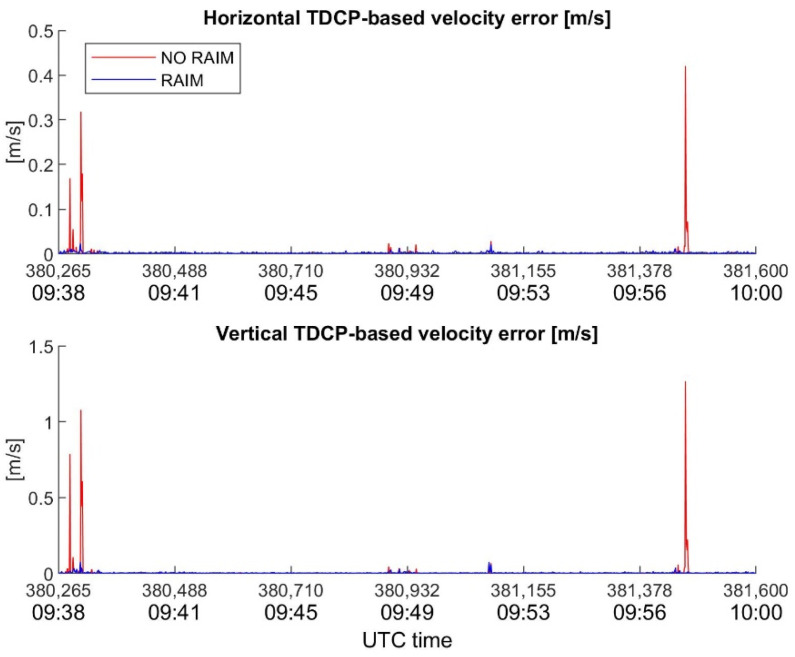
u-blox horizontal and vertical TDCP-based velocity errors as function of time, with and without RAIM application.

**Figure 12:**
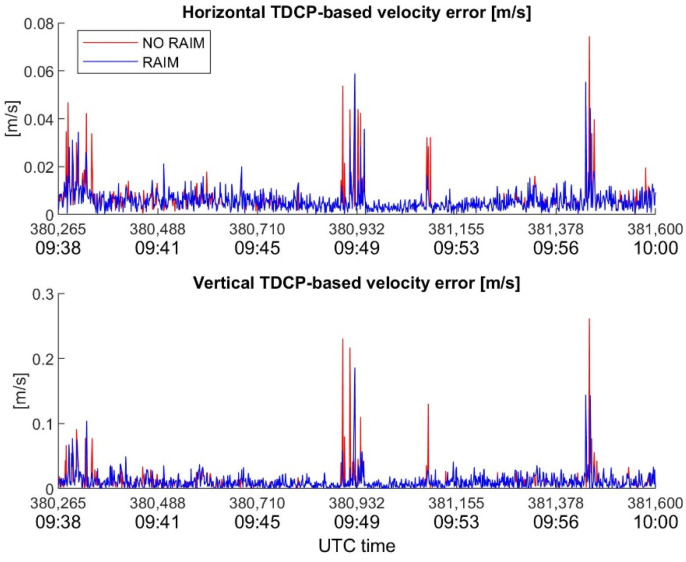
Xiaomi Mi8 horizontal and vertical TDCP-based velocity error as function of time, with and without RAIM application.

In order to underline the key role of RAIM in velocity estimation with TDCP, the behavior of velocity errors, with and without RAIM, for the considered devices is plotted in Figure 10, Figure 11 and Figure 12. It is interesting to note how the RAIM application is particularly effective for the high-sensitivity receiver; in fact, errors in the order of several dm/s for the horizontal channel, and of about one m/s for the vertical channel, are significantly reduced. However, RAIM is less effective for the Xiaomi Mi8, as is evident in Figure 12, where several spikes escape the RAIM check; the reason for this is the noisy measurements, which make it difficult for RAIM to work. Nevertheless, the benefits of RAIM to the Xiaomi case are evident, with a reduction in maximum errors of about 1.5 cm/s (horizontal) and 7.5 cm/s (vertical).

## 6. Conclusions

The objective of this study was to assess the performance of the TDCP technique, compared to the Doppler-based technique, for velocity estimation, with particular reference to smartphone GNSS chips. The motivation for this is related to the noisy observations characterizing such devices, implying degraded performance compared with other receivers, such as geodetic or consumer-grade receivers with dedicated antennas.

A static test was carried out in an open sky environment, using a geodetic-grade receiver with a survey antenna, a high-sensitivity receiver with a patch antenna, and a smartphone GNSS chip (Xiaomi Mi8). A RAIM algorithm was used in the data processing, to avoid the cycle slip issue which affects TDCP technique.

From the test, it is evident that TDCP is a valid approach for a precise velocity estimation for all the used devices, showing better performance than the Doppler-based approach. The geodetic receiver provided the best performance with RMS horizontal and vertical errors of about 2 and 4 mm/s, respectively, and a reliability percentage of 95.6%. The high-sensitivity receiver provided a slightly degraded performance compared with the geodetic receiver, with RMS horizontal and vertical errors of about 3 and 6 mm/s, respectively, and a similar reliable availability.

The Xiaomi Mi8 GNSS chip provided lower performance, with RMS horizontal and vertical errors of about 7 and 16 mm/s, respectively, and with a reliable availability of about 89%. The TDCP performance degradation of the smartphone GNSS chip compared with higher-grade devices confirms the typical observable noisiness of such types of devices, specifically of the carrier phase. Nevertheless, the maximum errors for the smartphone GNSS chip are not greater than 6 cm/s. Such performance allows the usage of TDCP-based velocity in advanced algorithms. RAIM plays an important role, significantly reducing the maximum errors. The reduced reliable availability suggests its usage in integrated systems (with inertial sensors for instance); a multi-GNSS approach could mitigate this problem.

## Figures and Tables

**Table 1:** Error analysis considering TDCP and Doppler techniques for Novatel Geodetic Receiver, u-blox high-sensitivity receiver, and Xiaomi Mi8 smartphone chip; RAIM is not applied.

Device	Technique	Mean [m/s]		RMS [m/s]		Max [m/s]		Solution Availability [%]
		H	U	H	U	H	U	
Novatel	Doppler	0.0176	0.0323	0.0202	0.0403	0.0567	0.1408	100
	TDCP	0.0018	0.0033	0.0021	0.0046	0.0181	0.0484	99.9
u-blox	Doppler	0.0264	0.0434	0.0306	0.0568	0.0952	0.2553	100
	TDCP	0.0037	0.0084	0.0179	0.0586	0.4204	1.2666	99.9
Xiaomi Mi8	Doppler	0.0151	0.0626	0.0202	0.0769	0.2813	0.8679	100
	TDCP	0.0060	0.0114	0.0083	0.0199	0.0744	0.2613	99.9

**Table 2:** Error analysis considering TDCP and Doppler techniques for Novatel Geodetic Receiver, u-blox high-sensitivity receiver, and Xiaomi Mi8 smartphone chip; RAIM is applied.

Device	Technique	Mean [m/s]		RMS [m/s]		Max [m/s]		Solution Reliability [%]
		H	U	H	U	H	U	
Novatel	Doppler	0.0176	0.0323	0.0202	0.0403	0.0567	0.1408	100
	TDCP	0.0017	0.0032	0.0020	0.0040	0.0131	0.0288	95.6
u-blox	Doppler	0.0264	0.0434	0.0306	0.0568	0.0952	0.2553	100
	TDCP	0.0023	0.0039	0.0029	0.0062	0.0238	0.0760	96.4
Xiaomi Mi8	Doppler	0.0151	0.0626	0.0202	0.0769	0.2813	0.8679	100
	TDCP	0.0055	0.0103	0.0071	0.0161	0.0589	0.1860	89.5

## Data Availability

The data presented in this study are available on request from the corresponding author.

## References

[1] Kaplan E.D., Hegarty C.J. (2006). Understanding GPS: Principles and Applications.

[2] Hofmann-Wellenhof B., Lichtenegger H., Collins J. (1992). GPS Theory and Practice Third Revised Edition.

[3] Tarig A. (2012). Positioning with wide-area GNSS networks: Concept and application. Positioning.

[4] U.S. GPS.GOV-Official U.S. Government Information about the Global Positioning System (GPS) and Related Topics. https://www.gps.gov/systems/gps/space/.

[5] Biagi L. (2006). I Fondamentali del GPS.

[6] Siddakatte R., Broumandan A., Lachapelle G. Performance evaluation of smartphone GNSS measurements with different antenna configurations. Proceedings of the International Navigation Conference.

[7] Fortunato M., Critchley-Marrows J., Siutkowska M., Ivanovici M.L., Benedetti E., Roberts W. Enabling high accuracy dynamic applications in urban environments using PPP and RTK on android multi-frequency and multi-GNSS smartphones. Proceedings of the 2019 European Navigation Conference (ENC).

[8] Pesyna K.M., Heath R.W., Humphreys E.T. Centimeter positioning with a smartphone-quality GNSS antenna. Proceedings of the 27th International Technical Meeting of the Satellite Division of The Institute of Navigation (ION GNSS+ 2014).

[9] van Graas F., Soloviev A. (2004). Precise velocity estimation using a stand-alone GPS receiver. Navigation.

[10] Petovello M., Gaglione S. (2015). How does a GNSS receiver estimate velocity?. Inside GNSS.

[11] Freda P., Angrisano A., Gaglione S., Troisi S. (2015). Time-differenced carrier phases technique for precise GNSS velocity estimation. Gps Solut..

[12] Soon B.K.H., Scheding S., Lee H.-K., Lee H.-K., Durrant-Whyte H. (2008). An approach to aid INS using time-differenced GPS carrier phase (TDCP) measurements. Gps Solut..

[13] Ding W., Wang J. (2011). Precise Velocity Estimation with a Stand-Alone GPS Receiver. J. Navig..

[14] Angrisano A., Vultaggio M., Gaglione S., Crocetto E.N. Pedestrian localization with PDR supplemented by GNSS. Proceedings of the 2019 European Navigation Conference (ENC).

[15] Zangenehnejad F., Gao Y. (2021). GNSS smartphones positioning: Advances, challenges, opportunities, and future perspectives. Satell Navig..

[16] Gikas V., Perakis E.H. (2016). Rigorous performance evaluation of smartphone GNSS/IMU sensors for ITS applications. Sensors.

[17] Liu W., Shi X., Zhu F., Tao X., Wang E.F. (2019). Quality analysis of multi-GNSS raw observations and a velocity-aided positioning approach based on smartphones. Adv. Space Res..

[18] Li Y., Wang L., Liu J., Zhang P., Lu E.Y. (2022). Accuracy Evaluation of Multi-Gnss Doppler Velocity Estimation Using Android Smartphones. Int. Arch. Photogramm. Remote Sens. Spat. Inf. Sci..

[19] Zhang X., Tao X., Zhu F., Shi X., Wang F. (2018). Quality assessment of GNSS observations from an Android N smartphone and positioning performance analysis using time-differenced filtering approach. Gps Solut..

[20] Guo L., Wang F., Sang J., Lin X., Gong X., Zhang W. (2020). Characteristics analysis of raw multi-GNSS measurement from Xiaomi Mi 8 and positioning performance improvement with L5/E5 frequency in an urban environment. Remote Sens..

[21] Robustelli U., Baiocchi V., Pugliano G. (2019). Assessment of dual frequency GNSS observations from a Xiaomi Mi 8 Android smartphone and positioning performance analysis. Electronics.

[22] Innac A., Angrisano A., Dardanelli G., della Corte V., Martellato E., Rotundi A., Ferraioli G., Palumbo P., Gaglione S. (2021). A Kalman Filter Single Point Positioning for maritime applications using a smartphone. Geogr. Tech..

[23] (2019). Ublox, ANN-MB Series Multi-Band, High Precision GNSS Antennas. Data Sheet. https://www.u-blox.com/sites/default/files/ANN-MB_DataSheet_(UBX-18049862).pdf.

[24] Tay S., Marais J. Weighting models for GPS Pseudorange observations for land transportation in urban canyons. Proceedings of the 6th European Workshop on GNSS Signals and Signal Processing.

[25] Parkinson B.W., Spilker J.J.J. (1996). Global Positioning System: Theory and Applications.

[26] Kirkko-Jaakkola M., Traugott J., Odijk D., Collin J., Sachs G., Holzapfel F. A RAIM approach to GNSS outlier and cycle slip detection using L1 carrier phase time-differences. Proceedings of the 2009 IEEE Workshop on Signal Processing Systems.

[27] ICAO (2018). Annex 10 to the Convention on International Civil Aviation-Aeronautical Telecommunications.

[28] Kuusniemi H. (2005). User-Level Reliability and Quality Monitoring in Satellite-Based Personal Navigation. Ph.D. Thesis.

[29] EUSPA (2018). World’s First Dual-Frequency GNSS Smartphone Hits the Market. https://www.euspa.europa.eu/newsroom/news/world-s-first-dual-frequency-gnss-smartphone-hits-market.

[30] Angrisano A., Gaglione S., Crocetto N., Vultaggio M. (2020). PANG-NAV: A tool for processing GNSS measurements in SPP, including RAIM functionality. GPS Solut..

